# Concurrent live imaging of DNA double-strand break repair and cell-cycle progression by CRISPR/Cas9-mediated knock-in of a tricistronic vector

**DOI:** 10.1038/s41598-018-35642-7

**Published:** 2018-11-23

**Authors:** Kensuke Otsuka, Masanori Tomita

**Affiliations:** Radiation Safety Research Center, Nuclear Technology Research Laboratory, Central Research Institute of Electric Power Industry (CRIEPI), Tokyo, 201-8511 Japan

## Abstract

Cell-cycle progression can be arrested by ionizing radiation-induced DNA double-strand breaks (DSBs). Although DSBs are patched by DSB repair systems, which comprise proteins such as p53-binding protein 1 (53BP1), the relationship between DSB repair progression and cell-cycle status in living cells is unclear. The probe FUCCI (fluorescent ubiquitination-based cell-cycle indicator) was previously developed for visualizing cell-cycle status. Here, we established novel live-imaging probes based on custom-designed plasmids designated “Focicles” harboring a tricistronic compartment encoding distinct fluorescent proteins ligated to the murine 53BP1 foci-forming region (FFR) and two cell-cycle indicators that are known components of FUCCI (hCdt1 and hGmnn). We used CRISPR/Cas9-mediated genome editing to obtain Focicle knock-in cell lines in NIH3T3 cells, which were subject to X-ray irradiation that induced comparable numbers of Focicle and endogenous-53BP1 foci. In addition, the Focicle probes enabled the kinetic analysis of both DSB repair and cell-cycle arrest/progression after irradiation, demonstrating that the Focicle knock-in cells progressed to cell division after DNA damage elimination. These newly developed probes can help to gain a better understanding of the dynamics of DSB repair and cell-cycle control to in turn guide cancer treatment development and cancer-risk assessments.

## Introduction

Visualization of intracellular molecules through fluorescent live imaging is a powerful technique for uncovering the biological dynamics of cells^1–3^. Specifically, live-cell imaging performed using recombinant fusion proteins labeled with fluorescent proteins (FPs) is the most widely employed method for elucidating the functions of various molecules of interest. For example, the status of the cell cycle can be visualized using the probe fluorescent ubiquitination-based cell-cycle indicator (FUCCI), which comprises two distinct FPs fused to the functional regions of cell cycle-specific molecules (hCdt1 and hGmnn)^4^. Cells typically progress along their programmed cell cycle; however, the cell cycle is occasionally arrested at certain phases due to the activation of signaling molecules such as ataxia telangiectasia mutated (ATM), p53, and p21^5–8^; these molecules are activated by certain physical stresses such as ionizing radiation, which efficiently generate DNA double-strand breaks (DSBs) in a dose-dependent manner. Most of the generated DSBs are effectively patched by the DSB-repair system, coordinated by the orchestrated action of DSB-repair proteins such as phosphorylated histone H2AX (*γ*-H2AX)^9^ and tumor-suppressor p53-binding protein 1 (53BP1)^10^. However, the spatiotemporal relationship between the progression of DSB repair and the cell-cycle status in living cells remains incompletely understood^8^. DSB sites can visualized based on the detection of DSB foci, which contain accumulated DSB-repair proteins. However, live imaging of *γ*-H2AX cannot be used for this purpose because the formation of *γ*-H2AX foci is triggered by its phosphorylation. By contrast, 53BP1 is widely recognized as a useful indicator for the live imaging of the DSB repair process, because the protein is recruited to methylated DNA histones and accumulates around DSB sites^11^. Moreover, 53BP1 recruitment at DSB sites is mediated by foci-forming regions (FFRs), including the Tudor domain^12^. This knowledge has facilitated the visualization of DSB-repair sites by using FPs fused to the FFR of 53BP1^13^.

Here, we developed a new live-imaging system using custom-designed plasmids named “Focicle” (“foci” + “cell cycle”), which harbored a tricistronic cassette encoding the FFR of mouse 53BP1 (m53BP1FFR) and two cell-cycle indicators (hCdt1 and hGmnn) fused to distinct FPs. We also established Focicle-knock-in cell lines in which the constructs were inserted at the ROSA26 locus, a well-known mouse safe-harbor site^14^, using CRISPR/Cas9-mediated genome editing. The CRISPR/Cas9 system is a well-established genome editing tool that can be used to cut double-stranded DNA at any site of choice^15,16^. Compared to conventional cloning techniques, CRISPR/Cas9-mediated targeting of the ROSA26 locus offers the advantage of avoiding potential interference of endogenous gene expression caused by random DNA integration^17^. Using this approach, we evaluated the progression of DSB repair and the cell-cycle status in Focicle knock-in cells after exposure to ionizing radiation.

## Results

### Design of tricistronic Focicle probes

First, we constructed three tricistronic plasmid vectors (Supplementary Fig. S1), each containing three inserts connected by self-cleaving 2A peptides (P2A or T2A). All inserts encode fusion proteins composed of the two cell-cycle indicators (hCdt1 and hGmnn) and m53BP1FFR (Supplementary Table S1 and Supplementary Methods) each connected to an FP. The DNA sequences used for both hCdt1 and hGmnn were selected according to the minimum regions identified to be required for protein function^4^. The sequence of m53BP1FFR was obtained from cDNA of mouse colonic cells (Supplementary Fig. S2). Each gene fragment was connected to generate a gene encoding three fusion FPs through seamless cloning and then assembled in a plasmid. For example, Focicle1 was designed to express Ypet/m53BP1FFR, mRuby3/hCdt1, and mTagBFP2/hGmnn. The other two plasmids used in this study, i.e., Focicle2 and Focicle3, were constructed in a similar manner (see Supplementary Fig. S1 and Supplementary Methods).

### CRISPR/Cas9-mediated knock-in of Focicle probe at the mouse ROSA26 locus and positional effects of the fusion proteins in the inserts

**Figure 1:**
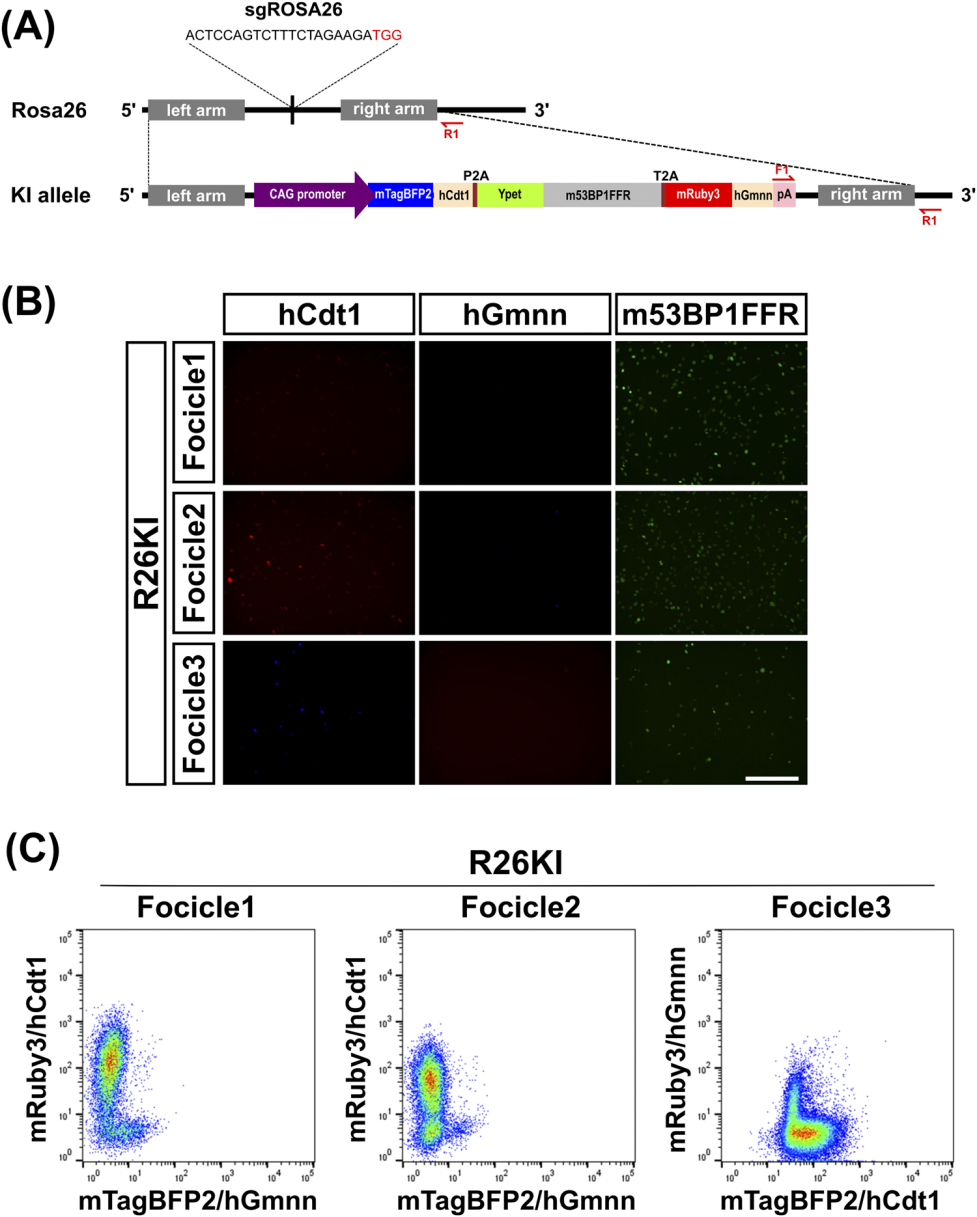
Construction of Focicle probe for CRISPR/Cas9-mediated knock-in at the ROSA26 locus. (**A**) Design of the Focicle probe. We constructed three pUC19-based plasmid vectors containing tricistronic inserts downstream of the CAG promoter in distinct orders: Focicle1: Ypet/m53BP1FFR, mRuby3/hCdt1, and mTagBFP2/hGmnn, connected by T2A and P2A self-cleaving peptides, respectively; Focicle2: mRuby3/hCdt1, Ypet/m53BP1FFR, and mTagBFP2/hGmnn, connected by P2A and T2A; and Focicle3: mTagBFP2/hCdt1, Ypet/m53BP1FFR, and mRuby3/hGmnn, connected by P2A and T2A peptides. The construction method used for R26KI-Focicle3 is depicted schematically. F1 and R1 denote the locations of the specific PCR primers used. (**B**) Fluorescence images showing the protein expression of hCdt1, hGmnn, and m53BP1 in NIH3T3 cells harboring R26KI-Focicle1, R26KI-Focicle2, and R26KI-Focicle3, respectively. Scale bar = 200 *μ*m. (**C**) Flow cytometry scattergrams of NIH3T3 cells harboring R26KI-Focicle1, R26KI-Focicle2, and R26KI-Focicle3.

Next, we established two plasmids for knock-in at the ROSA26 locus by means of genome editing. As a targeting vector, we constructed the pUC19-based Focicle-probe vector, sandwiched by mouse ROSA26 left- and right-arm sequences (Supplementary Fig. 3A). We also constructed a plasmid that concurrently expressed Cas9/NLS protein, sgRNA for targeting the mouse ROSA26 locus, and a marker FP (Supplementary Fig. 3B). Supplementary Figure 4 shows the scheme used for isolating the targeted cells after the knock-in was performed using these two plasmids. Based on the co-transfection of Focicle and Cas9-sgRNA plasmids, we ultimately isolated Focicle knock-in constructs harboring the tricistronic genes at the ROSA26 locus (Fig. 1A). Hereafter, these constructs are referred to as “R26KI-Focicle” constructs. The fluorescence intensity (FI) of Ypet/m53BP1FFR and mRuby3/hCdt1 in NIH3T3 cells harboring R26KI-Focicle1 was adequately high for detection under a conventional fluorescence microscope, whereas the fluorescence of mTagBFP2/hGmnn could not be readily detected (Fig. 1B). Similarly, in NIH3T3 cells harboring R26KI-Focicle2, the FI of mRuby3/hCdt1 and Ypet/m53BP1FFR was sufficiently high, but that of mTagBFP2/hGmnn was not. Although the design scheme for R26KI-Focicle3 and R26KI-Focicle2 were similar, mRuby3 was connected to hGmnn and placed at the 3^rd^ position, and mTagBFP2 was connected to hCdt1 and placed at the 1^st^ position (Supplementary Fig. S1). The FI of mTagBFP2/hCdt1 in NIH3T3 cells harboring R26KI-Focicle3 was slightly higher than that in cells harboring R26KI-Focicle2, whereas the FI of mRuby3/hGmnn was slightly diminished (Fig. 1B). The relative positional effects of the inserts were quantified by comparing the FI values obtained for these FP-fusion proteins with flow cytometry (Fig. 1C). As expected, we also confirmed that most of the FPs were localized in the nucleus.

### R26KI-Focicle probes enable the live imaging of DSB repair after radiation exposure

**Figure 2:**
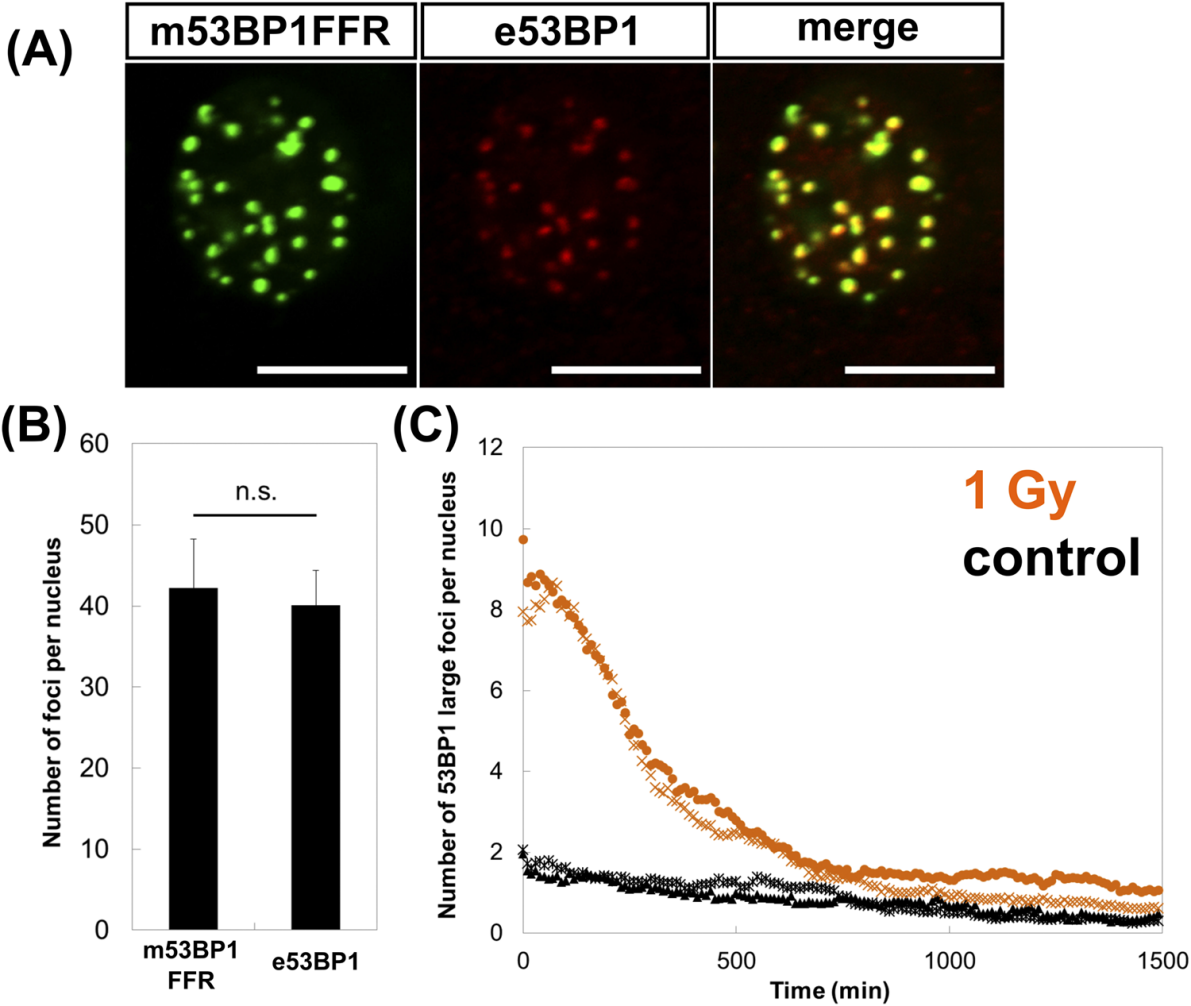
Formation of 53BP1 foci and repair kinetics after exposure to ionizing radiation. (**A**) Concomitant detection of a fluorescent protein (Ypet/m53BP1FFR) and of endogenous 53BP1 through immunofluorescent staining of NIH3T3 cells harboring R26KI-Focicle2, at 30 min after 1-Gy irradiation. Scale bar = 10 *μ*m. (**B**) Comparison of the numbers of Ypet/m53BP1FFR and endogenous 53BP1 foci (e53BP1) (n = 10). (**C**) Time-lapse imaging of DNA-damage repair in terms of 53BP1 large foci after 1-Gy X-ray irradiation (n = 2).

To determine whether our constructs can be used for detecting DSB repair, we examined the formation of foci in R26KI-Focicle-harboring NIH3T3 cells after radiation exposure. As expected, Ypet^+^ nuclear foci were observed after radiation exposure (Fig. 2A), with 42.2 ± 6.1 foci/Gy (n = 10) detected at 30 min after irradiation (Fig. 2B); this was comparable to the number of endogenous 53BP1 foci detected (40.1 ± 4.3/Gy, n = 10) (Fig. 2A,B). These results indicated that our constructs can be used for determining the precise numbers of 53BP1 foci. However, precise counting of foci requires high-resolution images taken under a high-magnification lens (e.g., 40× lens) and z-stack projection in each frame. Unfortunately, the microscope used in this study required a long time to capture each frame by high-magnification and z-stack projection, resulting in excessive laser exposure, making it difficult to perform quantitative data analysis. By contrast, we could obtain a large amount of data when capturing images under low magnification (20×) without z-stack projection. The trade-off of this approach was that smaller foci (lower than 2 pixels) could not be readily detected by the image analysis software. Based on these technical limitations, for evaluation of the validity of our probe in time-lapse live-cell imaging, we captured all images under low magnification (20×) and then counted the number of only large foci per nucleus (≥2 *μ*m) through image analysis. Figure 2C shows the repair kinetics of 53BP1 large foci per nucleus after radiation exposure: the number of large foci increased immediately after 1-Gy irradiation and then gradually decreased to approach the background level within 10 h after irradiation.

### Validation of Focicle as a cell-cycle indicator

**Figure 3:**
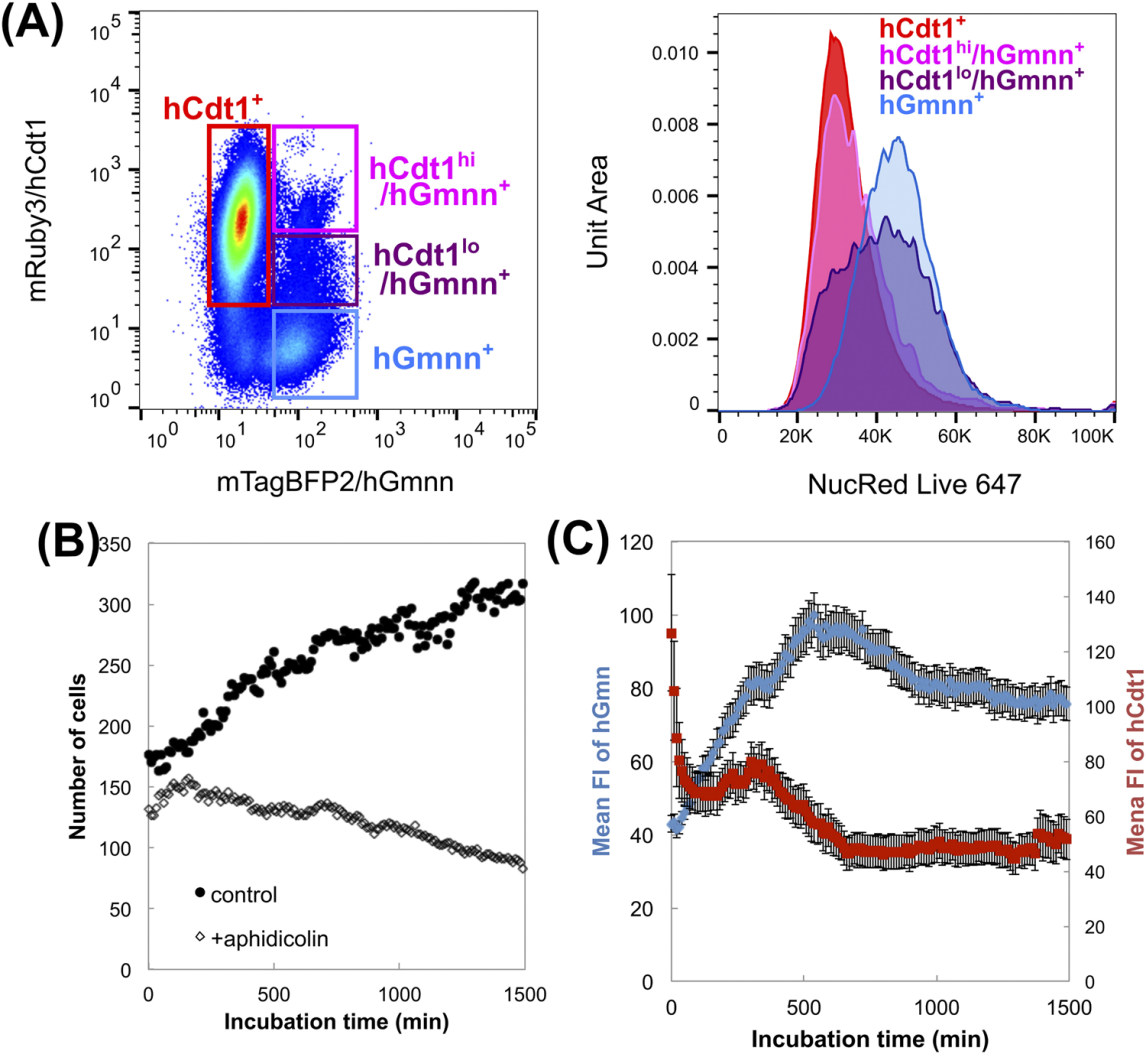
Validation of cell-cycle indicators. (**A**) A flow cytometric pseudocolor plot of confluent NIH3T3 cells harboring R26KI-Focicle2 (left, figure). Four gates were set: hCdt1^+^ (mRuby3^+^/mTagBFP2^−^), hCdt1^hi^/hGmnn^+^ (mRuby3^hi^/mTagBFP2^+^), hCdt1^lo^/hGmnn^+^ (mRuby3^lo^/mTagBFP2^+^), and hGmnn^+^ (mRuby3^−^/mTagBFP2^+^), and the DNA contents of the cells in each gate are shown per unit area (right, histogram). (**B**,**C**) Aphidicolin-induced cell-cycle arrest estimated based on (**B**) the number of cells (nuclei) per frame and (**C**) the mean fluorescence intensity (FI) of hGmnn (left axis) and hCdt1 (right axis); means ± SEM are shown.

To validate the use of R26KI-Focicle probes as cell-cycle indicators, the DNA contents of the hCdt1^+^/hGmnn^−^, hCdt1^hi^/hGmnn^+^, hCdt1^lo^/hGmnn^+^, and hCdt1^−^/hGmnn^+^ fractions were evaluated by flow cytometry (Fig. 3A). The distribution of the DNA contents gradually increased from the hCdt1^hi^/hGmnn^+^ to the hCdt1^−^/hGmnn^+^ gate (Fig. 3B). Moreover, in R26KI-Focicle-harboring NIH3T3 cells, the mean FI of nuclear hGmnn increased over time and then the fluorescence disappeared abruptly at the point of maximal intensity (Supplementary Video S1). To synchronize the cell-cycle status, we treated the cells with aphidicolin, an inhibitor of DNA replication in S phase, which resulted in growth inhibition of NIH3T3 cells harboring R26KI-Focicle (Fig. 3B). The FI of hGmnn gradually increased to a high level after the addition of aphidicolin, whereas that of hCdt1 decreased to a low level, indicating cell-cycle arrest at the S/G2 phase (Fig. 3C).

### Concurrent detection of DSB foci and cell-cycle status

**Figure 4:**
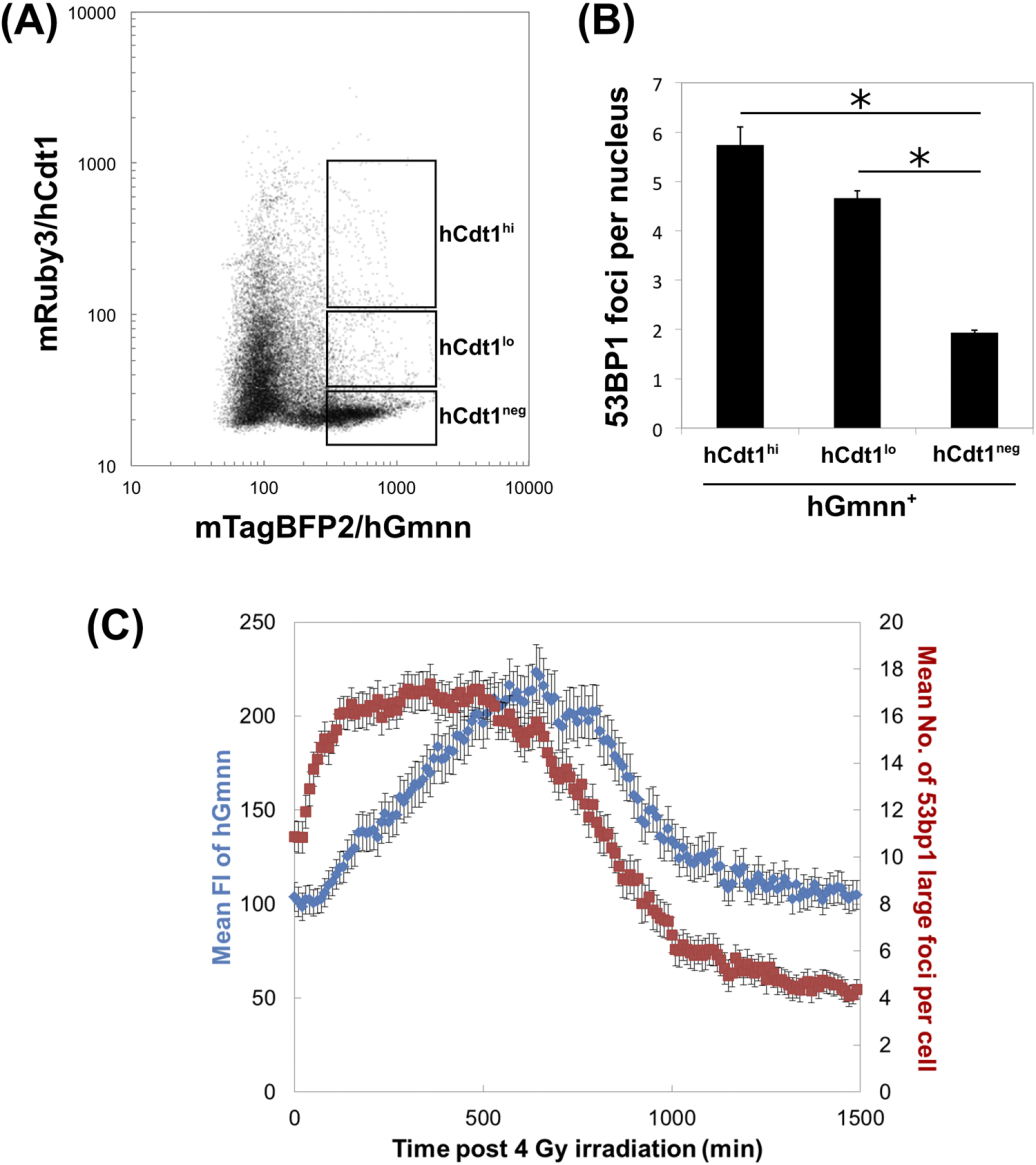
Concurrent quantification of cell-cycle indicators and analysis of DSB-repair kinetics. (**A**) Two-dimensional plot for fluorescence intensity (FI) of mTagBFP2/hGmnn and mRuby3/hCdt1 in growing NIH3T3 cells harboring R26KI-Focicle2 without irradiation. Image analysis was applied to all frames of time-lapse live-cell imaging. All data (22,118 points) were plotted according to the FIs of mRuby and mTagBFP2. To evaluate the number of 53BP1 foci during DNA synthesis, three gates, i.e., high level of hCdt1 expression (hCdt1^hi^), low level of hCdt1 (hCdt1^lo^), and cells without hCdt1 expression (hCdt1^neg^), were determined in the hGmnn^+^ population. (**B**) Number of 53BP1FFR large foci in the gates of (**A**); mean ± SEM. *p < 0.01. (**C**) Concomitant analysis of DSB-repair kinetics and cell-cycle status after 4-Gy irradiation. Left axis (blue diamonds): mean FI of hGmnn; right axis (red squares): number of 53BP1 large foci per nucleus. Means ± SEM are shown.

The results described above indicated that our probes could be successfully used for evaluating both 53BP1 foci and cell-cycle progression independently. Thus, we further analyzed the correlation between the number of foci and cell-cycle progression under several culture conditions to evaluate the feasibility of their simultaneous evaluation. First, to understand the steady-state foci dynamics, we evaluated the relationship of three fluorescent proteins in non-irradiated NIH3T3 cells harboring R26KI-Focicle. Figure 4A shows a dot plot of the FI for both mTagBFP2/hGmnn and mRuby3/hCdt1 in NIH3T3 cells harboring R26KI-Focicle2. We applied image analysis with commercial software to capture all frames of time-lapse live-cell imaging (150 frames), which were plotted with all of the cell data in Fig. 4A (22,118 data points). Thus, each data point in Fig. 4A contains information about the number of 53BP1 foci as well as the FIs of hCdt1/hGmnn. We placed the three gates of the hGmnn^+^ population along with its hCdt1^+^ expression status, i.e., high-level expression of hCdt1 (hCdt1^hi^), low-level expression (hCdt1^lo^), and hGmnn alone hCdt1^neg^, to compare the number of 53BP1 foci in the early S, late S, and G2 phase, respectively. Figure 4B shows the mean number of 53BP1FFR foci in the hCdt1^hi^, hCdt1^lo^, and hCdt1^neg^ groups among hGmnn^+^ cells. The number of foci significantly decreased along with cell-cycle progression (p < 0.01, Scheffe’s multiple comparison), suggesting that the cells could progress to cell division once most of the DNA damage had been repaired. To evaluate whether cell-cycle progression proceeds after the disappearance of 53BP1 foci following exposure to ionizing radiation, we performed time-lapse live-cell imaging of NIH3T3 cells harboring R26KI-Focicle2 after 4 Gy of acute X-ray irradiation. After irradiation, hGmnn molecules accumulated in accordance with the formation of 53BP1 foci, and, most notably, the cell-cycle arrest was gradually released following DSB repair (Supplementary Video S2, Fig. 4C). These results provide evidence as to how cells handle DNA damage and control the timeline of cell-cycle progression in the face of genotoxic stresses.

## Discussion

We developed three plasmids encoding probes that enable the concurrent live imaging of DSB repair and cell-cycle status. For establishing the knock-in cell lines, the following three criteria were applied: the cells should (1) be generated readily and quickly, (2) be useful for diverse research purposes, and (3) retain a neutral phenotype. In previous studies, a two-step transfection protocol was necessary for isolating cells harboring both 53BP1/FP and FUCCI. However, with our newly developed method, target cells can be obtained through a single transfection step, because all inserts are encoded by a single transcript. We achieved this by carefully considering the order and combination of the tricistronic genes in the vectors, because protein expression levels could be altered when the coding genes are connected by 2A peptides.

A systematic comparison of multiple genes connected by 2A peptide-encoding sequences in tricistronic vectors revealed that protein expression levels were the highest for the gene at the 1^st^ position, relatively lower for the gene at the 3^rd^ position, and lowest for the gene at the 2^nd^ position^18^. Therefore, it is crucial to consider the position of the FPs in a probe. We also constructed a plasmid harboring hGmnn at the 1^st^ position, which showed sufficiently strong fluorescent intensity; however, the fluorescence expression was cell cycle-independent because hGmnn had to be placed at C-terminus, which is expected to function as a cell-cycle indicator^19^. The results of long-term, time-lapse live-cell imaging under a fluorescence microscope indicated that it is critical to reduce the phototoxic damage of cells and the bleaching of FPs caused by overexposure to the light source^20^. To avoid this drawback, we selected some of the brightest available FPs: mTagBFP2^21^, Ypet^22^, mRuby3^23^, and iRFP670^24^, for blue, yellow-green, red, and far-red FPs, respectively. We did not select green fluorescent protein (GFP), as this is the most widely used FP in cellular and molecular imaging analysis, and thus its inclusion could potentially interfere with other applications. Furthermore, because we used the CRISPR/Cas9 technique, we were typically able to obtain the knock-in cells within one week. Given that our strategy is based on a one-step knock-in strategy performed using the CRISPR/Cas9 system, the designed plasmids can be used with not only cell lines but also with primary cultured cells. Notably, primary cells can be cultured to form three-dimensional organ-like structures, which are now well established as organoids. Thus, a major advantage offered by our strategy is its usability when mimicking organs, which can facilitate the clinical translation of the method.

Radiation-induced DSBs increase in a dose-dependent manner. In general, the number of DSBs could be accurately represented by the number of *γ*-H2AX foci. Cariveaua *et al*.^25^ evaluated the appearance and disappearance of *γ*-H2AX foci in NIH3T3 cells after 2- and 4-Gy irradiation, and found that the number of *γ*-H2AX foci reached a maximum at 0.5 h after irradiation. They also showed a dose-dependent delay of the disappearance of *γ*-H2AX foci, which is line with the dynamics of foci disappearance observed in the present study. The maximum yields of *γ*-H2AX foci are estimated at approximately 35 per Gy in human cells^26^. Consistently, we determined that there were approximately 40 foci per nucleus based on images obtained by the microscope with z-stack and maximum projection. However, the status of the cell cycle should be taken into account whenever counting the number of foci, because the numbers of DSBs is proportional to the DNA content^27^, which changes during cell-cycle progression; therefore, the DSB yield depends on the cell-cycle status^28^. Variation in foci estimation is recognized as a common problem in radiation research. Belyaev summarized the problems in foci enumeration^29^. For instance, the threshold of intensity^30^, magnification of images^31^, interlaboratory differences in criteria, cell culture conditions, cell-cycle phase, and senescence^32^ have been recognized as important considerations in radiation-based research. The challenges are not only technical limitations but also physiological features of various cells. Although we encountered these problems, our technical advancement enabled us to proceed a kinetic analysis between the appearance/disappearance of DSB foci and the phase of cell cycle through basic research. Since our Focicle probe is a tool for live-cell imaging following gene knock-in at the safe-harbor site of mouse genome, it can be applied for validation of any mouse cell line under various culture conditions. Owing to technical limitations of the microscope used, in this assessment, we focused on the validity of the probes to evaluate the kinetics of large foci in correlation with cell-cycle status. The focus on large foci has biological significance because large foci around 2 *μ*m^2^ have been reported to play a role in triggering G1 arrest^28,33^. However, small foci are also of relevance, and it may be possible to count even small foci with a super-resolution microscope^34,35^. Therefore, in future work, we plan to find a compatible balance between counting all foci while still achieving high-throughput time-lapse imaging.

Elucidation of the kinetics of cell cycle-dependent DSB repair will provide information that can be useful for developing cancer treatments and for assessing cancer risk. Cancers are considered to originate from genetic mutations that are mainly derived from misrepaired DSBs, and thus failure of the DNA-damage response can lead to the development of cancer-prone genetic diseases. Reyes *et al*. recently found, by live-imaging of 53BP1 foci and hGmnn-FP, that the G1/S transition occurs even in cells with DNA damage caused by high-dose (20 Gy) radiation^36^. Our results showed that DSB repair is an important event for G2/M transition. Thus, the modification of cell cycle-specific DSB-repair proteins represents a potential target in anticancer therapy^37^. However, most chemoresistant and radioresistant cancer cells are known to exist in a quiescent state; thus, cell-cycle control is an important target in cancer treatment^38^.

In summary, we established R26KI-Focicle as a useful probe for elucidating the kinetics of both DSB repair and cell-cycle arrest/progression after radiation exposure in mouse samples. This general approach can also be applied to design constructs for human samples. Similar to the mouse ROSA26 locus, human adeno-associated virus integration site 1 (AAVS1) is widely recognized as a safe-harbor site in the human genome^39^. Based on this finding, we plan to establish Focicle probes suitable for human materials in future work.

## Methods

### Plasmids and transfection

The details of the construction of Focicle vectors are presented in the Supplementary Methods. In brief, the backbone vectors pCAG, pGuide-it, and pUC19L were purchased from Addgene (Cambridge, MA), Clontech Laboratories, Inc. (Mountain View, CA), and ThermoFisher Scientific Inc. (Waltham, MA), respectively, and the inserts were synthesized or isolated from cDNAs using conventional PCR and purified using agarose gel electrophoresis. Vectors harboring the inserts were constructed using a conventional DNA ligation kit (#6023, Takara Bio, Inc., Shiga, Japan), GeneArt Seamless Cloning and Assembly Kit (#A13288, ThermoFisher Scientific Inc.), or GeneArt Seamless PLUS Cloning and Assembly Kit (#A14603, ThermoFisher Scientific Inc.). The vectors were used to transform competent cells (TOP10 or DH10B T1SA) and then purified using a plasmid Midi kit (#12143, QIAGEN, Hilden, Germany).

### Cell line, transfection, and gene editing

NIH3T3 cells were purchased from American Type Cell Culture cell bank (#CRL-1658, #PN127) and cultured in Dulbecco’s modified essential medium containing 10% bovine serum (#16170078, ThermoFisher Scientific Inc.) and 1X penicillin/streptomycin (#15140148, ThermoFisher Scientific Inc.). Subcultured NIH3T3 cells were centrifuged (90 × g) and pellets (1 × 10^6^ cells) were resuspended in 100 *μ*L of mixing buffer of the SG cell line 4D-Nucleofector kit (#V4XC-3024, LONZA Ltd., Basel, Switzerland) together with plasmids (pUC19-R26L-CAG-Focicle-R26R and Cas9-sgROSA26-iRFP670), and the cells were then transfected by means of electroporation performed using 4D-Nucleofector (LONZA Ltd.) according to the manufacturer’s instructions, which was optimized for NIH3T3 cells (#EN-158). To examine and photograph live cells expressing FPs, we used a BZ-X710 fluorescence microscope (KEYENCE Corp., Osaka, Japan).

### Time-lapse live-cell imaging

For time-lapse imaging of FPs in living cells, we used a C1 confocal microscope (Nikon Corp., Tokyo, Japan) with a cell-culture unit, which enabled maintaining the stage at 37 °C with 5% CO_2_. Four channels (Blue, Green, Red, and Transmitted) of images (1024 × 1024 pixels) were captured every 10 min for up to 150 frames (25 h). Finally, image analysis was performed using the General Analysis function in Nikon’s NIS-Elements AR software (Ver. 4.40).

### Irradiation

Cells were X-ray irradiated, at 1 or 4 Gy, with an MBR-1520R4 system (Hitachi Ltd., Tokyo, Japan) operated at 150 kV, with a 20-mA tube current and a 0.5-mm Al + 0.3-mm Cu filter. The dose-rate was 0.6 Gy/min. We irradiated the cells at room temperature (24 °C). After irradiation, the cells were transferred to the culture unit of the confocal microscope. Preparation for time-lapse imaging took approximately 5 min after the irradiation terminated. Overall, it took approximately 10 to 15 min to obtain the first frame from the beginning of the irradiation.

### Cell sorting and flow cytometry

Flow cytometry data were analyzed and cells were isolated using a MoFlo Astrios EQ system (Beckman Coulter, Inc., Brea, CA), and FCS data were obtained using Summit Software (Beckman Coulter, Inc.) and then analyzed using the commercial software FlowJo version 10.2 (FlowJo, LLC, Ashland, OR). To determine the DNA contents in live cells, the cells were incubated in medium containing NucRed Live 647 ReadyProbes Reagent (#R37106, ThermoFisher Scientific Inc.), according to the manufacturer’s instructions.

### Immunofluorescent staining

To count the numbers of foci of endogenous mouse 53BP1, R26KI-Focicle knock-in cells were cultured on a glass-bottom cell imaging dish (#0030740009, Eppendorf AG, Humburg, Germany) and irradiated with X-rays (1 Gy). Thirty minutes after the irradiation, the cells were washed by phosphate buffered saline (PBS) and fixed with 4% paraformaldehyde (#163-20145, Wako Pure Chemical Industries, Ltd., Osaka, Japan) at 37 °C for 10 min, and placed in 70% ice-cold ethanol for 30 min. The samples were then washed with a wash buffer (2% bovine serum albumin and 0.05% Tween 20 dissolved in PBS) three times. Samples were permeabilized by 0.5% TritonX-100/PBS at 4 °C for 30 min, and then incubated with anti-53BP1 antibody (#A300-272A, Bethyl Laboratories, Inc., Montgomery, TX) dissolved in wash buffer at 4 °C overnight. The samples were washed with wash buffer three more times and incubated in Alexa647-conjugated 2^nd^ antibody (#A21244, ThermoFisher Scientific Inc.) dissolved in wash buffer at 4 °C for 1 h.

### Statistical analyses

The numbers of foci per nucleus were compared between two or three experimental groups using Student’s t-test or Scheffe’s multiple comparison test, respectively.

## Electronic supplementary material

Supplementary Information

Supplementary Video S1

Supplementary Video S2

## References

[1] Giepmans BN, Adams SR, Ellisman MH, Tsien RY (2006). The fluorescent toolbox for assessing protein location and function. Science.

[2] Lippincott-Schwartz J, Snapp E, Kenworthy A (2001). Studying protein dynamics in living cells. Nat. Rev. Mol. Cell Biol..

[3] Zhang J, Campbell RE, Ting AY, Tsien RY (2002). Creating new fluorescent probes for cell biology. Nat. Rev. Mol. Cell Biol..

[4] Sakaue-Sawano A (2008). Visualizing spatiotemporal dynamics of multicellular cell-cycle progression. Cell.

[5] Brugarolas J (1995). Radiation-induced cell cycle arrest compromised by p21 deficiency. Nat..

[6] Bunz F (1998). Requirement for p53 and p21 to sustain G2 arrest after DNA damage. Science.

[7] Dulic V (1994). p53-dependent inhibition of cyclin-dependent kinase activities in human fibroblasts during radiation-induced G1 arrest. Cell.

[8] Khanna KK, Jackson SP (2001). DNA double-strand breaks: signaling, repair and the cancer connection. Nat. Genet..

[9] Rogakou EP, Pilch DR, Orr AH, Ivanova VS, Bonner WM (1998). DNA double-stranded breaks induce histone H2AX phosphorylation on serine 139. J. Biol. Chem..

[10] Rappold I, Iwabuchi K, Date T, Chen J (2001). Tumor suppressor p53 binding protein 1 (53BP1) is involved in DNA damage-signaling pathways. J. Cell Biol..

[11] Schultz LB, Chehab NH, Malikzay A, Halazonetis TD (2000). p53 binding protein 1 (53BP1) is an early participant in the cellular response to DNA double-strand breaks. J. Cell Biol..

[12] Iwabuchi K (2003). Potential role for 53BP1 in DNA end-joining repair through direct interaction with DNA. J. Biol. Chem..

[13] Dimitrova N, Chen YC, Spector DL, de Lange T (2008). 53BP1 promotes non-homologous end joining of telomeres by increasing chromatin mobility. Nat..

[14] Soriano P (1999). Generalized lacZ expression with the ROSA26 Cre reporter strain. Nat. Genet..

[15] Cong L (2013). Multiplex genome engineering using CRISPR/Cas systems. Science.

[16] Jinek M (2012). A programmable dual-RNA-guided DNA endonuclease in adaptive bacterial immunity. Science.

[17] Chu VT (2016). Efficient generation of Rosa26 knock-in mice using CRISPR/Cas9 in C57BL/6 zygotes. BMC Biotechnol..

[18] Liu Z (2017). Systematic comparison of 2A peptides for cloning multi-genes in a polycistronic vector. Sci. Reports.

[19] Mort R (2014). Fucci2a: a bicistronic cell cycle reporter that allows Cre mediated tissue specific expression in mice. Cell Cycle.

[20] Boudreau C (2016). Excitation light dose engineering to reduce photo-bleaching and photo-toxicity. Sci. Reports.

[21] Subach OM, Cranfill PJ, Davidson MW, Verkhusha VV (2011). An enhanced monomeric blue fluorescent protein with the high chemical stability of the chromophore. PLoS One.

[22] Nguyen AW, Daugherty PS (2005). Evolutionary optimization of fluorescent proteins for intracellular FRET. Nat. Biotechnol..

[23] Bajar BT (2016). Improving brightness and photostability of green and red fluorescent proteins for live cell imaging and fret reporting. Sci. Reports.

[24] Shcherbakova DM, Verkhusha VV (2013). Near-infrared fluorescent proteins for multicolor *in vivo* imaging. Nat. Methods.

[25] Cariveaua M (2006). Correlations between radiation-induced double strand breaks and cell cycle checkpoints in X-irradiated nih/3t3 fibroblasts. Anticancer. Res..

[26] Rothkamm K, Lobrich M (2003). Evidence for a lack of DNA double-strand break repair in human cells exposed to very low x-ray doses. Proc. Natl. Acad. Sci. USA.

[27] Cedervall B (1995). Methods for the quantification of DNA double-strand breaks determined from the distribution of DNA fragment sizes measured by pulsed-field gel electrophoresis. Radiat. Res..

[28] Costes S (2010). Spatiotemporal characterization of ionizing radiation induced DNA damage foci and their relation to chromatin organization. Mutat. Res..

[29] Belyaev I (2010). Radiation-induced DNA repair foci: Spatio-temporal aspects of formation, application for assessment of radiosensitivity and biological dosimetry. Mutat. Res..

[30] Yasui L (2004). GammaH2AX foci induced by gamma rays and 125idU decay. Int. J. Radiat. Biol..

[31] Han J, Hendzel M, Allalunis-Turner J (2006). Quantitative analysis reveals asynchronous and more than DSB-associated histone H2AX phosphorylation after exposure to ionizing radiation. Radiat. Res..

[32] Nakamura A, Redon C, Bonner W, Sedelnikova O (2009). Telomere-dependent and telomere-independent origins of endogenous DNA damage in tumor cells. Aging.

[33] Yamauchi M (2008). Growth of persistent foci of DNA damage checkpoint factors is essential for amplification of G1 checkpoint signalling. DNA Repair.

[34] Hagiwara Y (2017). Spatiotemporal characterization of ionizing radiation induced DNA damage foci and their relation to chromatin organization. Oncotarget.

[35] Sisario, D. *et al*. Nanostructure of DNA repair foci revealed by superresolution microscopy. *FASEB J*. **32**, epub ahead (2018). 10.1096/fj.201701435 29894665

[36] Reyes J (2018). Fluctuations in p53 signaling allow escape from cell-cycle arrest. Mol. Cell.

[37] Gullotta F, De Marinis E, Ascenzi P, di Masi A (2010). Targeting the DNA double strand breaks repair for cancer therapy. Curr. Med. Chem..

[38] Hartwell LH, Kastan MB (1994). Cell cycle control and cancer. Science.

[39] Kotin RM, Linden RM, Berns KI (1992). Characterization of a preferred site on human chromosome 19q for integration of adeno-associated virus DNA by non-homologous recombination. EMBO J..

