# Supplementary material for: Concurrent live imaging of DNA double-strand break repair and cell-cycle progression by CRISPR/Cas9-mediated knock-in of a tricistronic vector
Source: Sci Rep. 2018 Nov 23;8:17309. doi: 10.1038/s41598-018-35642-7 (PMC6251881; doi:10.1038/s41598-018-35642-7)
Supplement: Supplementary file 1 — Supplementary Information [file 41598_2018_35642_MOESM1_ESM.docx]

**Supplementary Information**

Concurrent live imaging of DNA double-strand break repair and cell-cycle progression by CRISPR/Cas9-mediated knock-in of a tricistronic vector

Kensuke Otsuka^*^, Masanori Tomita

Radiation Safety Research Center, Nuclear Technology Research Laboratory, Central Research Institute of Electric Power Industry (CRIEPI), Tokyo, Japan

*Corresponding Author: Kensuke Otsuka, Radiation Safety Research Center, Nuclear Technology Research Laboratory, Central Research Institute of Electric Power Industry (CRIEPI)

Postal Address: 2-11-1 Iwado-kita, Komae, Tokyo 201-8511, Japan

Phone: +81-3-3480-2111; Fax: +81-3-3480-3113; E-mail: ohken@criepi.denken.or.jp

**Supplementary Methods**

*Plasmids and transformation*

pCAG-LSL-ZsGreen was purchased from Addgene (#51269) and was digested by two restriction enzymes (ThermoFisher Scientific): PacI (#FD2204) and SacI (#FD1133). Digested plasmids were purified by conventional agarose gel electrophoresis following column-based purification using NucleoSpin Gel and PCR Clean-Up (Macherey-Nagel Inc., #740609). The linearized pUC19 plasmid (pUC19L) vector (ThermoFisher Scientific) was used for assembling fusion genes. The linearized pGuide-it tdTomato vector was purchased from Clontech Laboratory (#632604). Insert genes were amplified by conventional PCR using Platinum SuperFi DNA polymerase (ThermoFisher Scientific, #12351-050) and specific primers (Supplementary Table S1), and then the PCR products were purified by conventional agarose gel electrophoresis following column-based purification using NucleoSpin Gel and PCR Clean-Up (Macherey-Nagel Inc., #740609). Fragments of hCdt1 (amino acids 30–120, 273 bp) and hGmnn (amino acids 1–110, 330 bp) were isolated from the cDNA of MOLT-4 and HEK-293T cells, respectively. The sequence of the mouse 53BP1 foci-forming region (m53BP1FFR) for live imaging was determined as the complementary sequence of the human 53BP1 FFR (Dimitrova et al., 2008), and the fragments were obtained from colonic cDNA of a C57BL/6J mouse by conventional PCR using specific primers (Supplementary Table S1) and Platinum SuperFi DNA polymerase. As the PCR templates, mRuby3, mTagBFP2, and Ypet genes, encoding red, blue, and green-yellow fluorescent protein, respectively, were synthesized by GeneArt Strings Service (ThermoFisher Scientific) and their PCR products for seamless cloning were obtained using specific primers (Supplementary Table S1). For construction of mRuby3/hCdt1, PCR products of mRuby3 and hCdt1 were cloned in the pUC19L vector by GeneArt Seamless Cloning and Assembly Kit (ThermoFisher Scientific, #A13288), and the vectors were transformed in TOP10 competent cells. Likewise, the PCR products of mTagBFP2 and hGmnn, and Ypet and mBP1TD were cloned in the pUC19L vector, which were transformed in TOP10 competent cells. The fragments of the three fusion genes were obtained by PCR using specific primers (Supplementary Table S1) and cloned in the pCAG vector, which was transformed in DH10B T1SA competent cells by GeneArt Seamless PLUS Cloning and Assembly Kit (ThermoFisher Scientific, #A14603). Plasmids for CRISPR/Cas9-mediated knock-in at the ROSA26 locus were generated by digesting pCAG tricistronic plasmids with the restriction enzymes (ThermoFisher Scientific) SgsI (#FD1894) and NotI (#FD0594), and assembled with pUC19 vector, ROSA26 left arm (R26L), and ROSA26 right arm (R26R) by Seamless Cloning (Supplementary Fig. S3A). The sgRNA sequence for targeting the ROSA26 locus (sgROSA26) was designed based on a previous study (Chu et al., 2016). The oligonucleotide of sgROSA26 was ligated to the pGuide-it tdTomato vector with the Mighty Mix DNA Ligation kit (Takara, #6023) and cloned in TOP10 competent cells, and the vector was purified by plasmid Midi kit (QIAGEN) to obtain Cas9-sgROSA26-tdTomato plasmid. To avoid overlapping of red fluorescence, we cut off the tdTomato sequence from the Cas9-sgROSA26-tdTomato plasmid by two restriction enzymes: EcoRI (#FD0274) and Bsu36I (#FD0374). The linearized Cas9-sgROSA26 vector was then ligated with iRFP670 to obtain the Cas9-sgROSA26-iRFP670 plasmid (Supplementary Fig. S3B).

*Knock-in of Focicle probe at the ROSA26 locus by CRISPR/Cas9-mediated gene editing*

Supplementary Fig. S4 shows the scheme used for generating R26KI-Focicle cells. The pUC19-R26L-Focicle-R26R plasmid and the Cas9-sgROSA26-iRFP670 plasmid were co-transfected into NIH3T3 cells by electroporation as described in the main text. To improve the recombination efficiency, we added 5 μm of L755507 (Xcess Biosciences, #M60082-2S) to the medium (Yu et al., 2015). On day 1, we isolated Ypet^+^/iRFP670^+^ cells by cell sorting to purify the cells that expressed fluorescence derived from the two types of plasmids (Supplementary Fig. S4). The Ypet^+^/iRFP670^+^ isolated cells were then cultured for several days in L755507-supplemented medium. We considered that the knock-in process was completed within one week and the expression of Cas9 protein was no longer needed at this stage. Thus, on day 6–7, we enriched the Ypet^+^/iRFP670^-^ population by cell sorting and continued subculture thereafter (Supplementary Figs. S4 and S5). To check whether the knock-in process had been performed properly, genomic DNA of the cells was extracted and the constructs were checked by PCR (Supplementary Fig. S6).

**References**

Chu, V.T., Weber, T., Graf, R., Sommermann, T., Petsch, K., Sack, U., Volchkov, P., Rajewsky, K. and Kuhn, R. (2016). Efficient generation of Rosa26 knock-in mice using CRISPR/Cas9 in C57BL/6 zygotes. BMC Biotechnol 16, 4.

Dimitrova, N., Chen, Y.C., Spector, D.L. and de Lange, T. (2008). 53BP1 promotes non-homologous end joining of telomeres by increasing chromatin mobility. Nature 456, 524-528.

Yu, C., Liu, Y., Ma, T., Liu, K., Xu, S., Zhang, Y., Liu, H., La Russa, M., Xie, M., Ding, S., et al. (2015). Small molecules enhance CRISPR genome editing in pluripotent stem cells. Cell Stem Cell 16, 142-147.

Zgheib, O., Pataky, K., Brugger, J. and Halazonetis, T.D. An oligomerized 53BP1 tudor domain suffices for recognition of DNA double-strand breaks. (2009). Mol Cell Biol 29, 1050-1058.

Fradet-Turcotte, A., Canny, M.D., Escribano-Diaz, C., Orthwein, A., Leung, C.C., Huang, H., Landry, M.C., Kitevski-LeBlanc, J., Noordermeer, S.M., Sicheri, F, and Durocher, D. (2013). 53BP1 is a reader of the DNA-damage-induced H2A Lys 15 ubiquitin mark. Nature 499, 50-54.

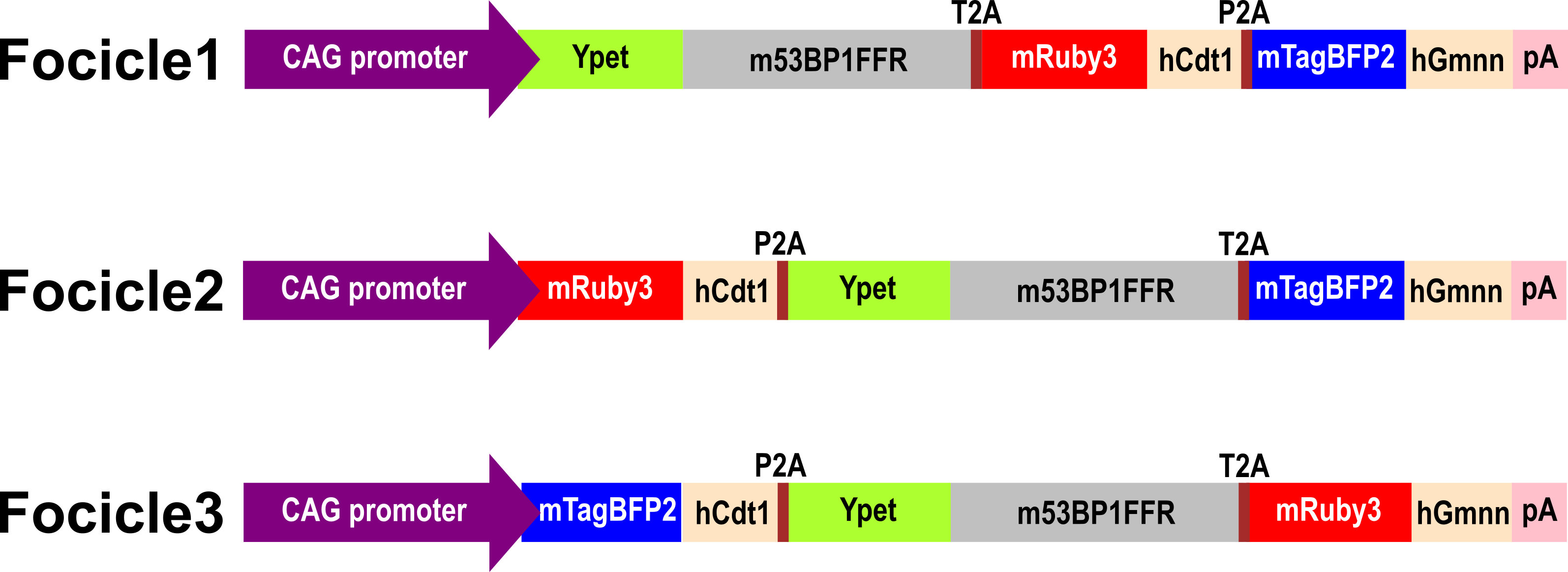


**Supplementary Fig. S1**

Design of Focicle probes. We developed three types of probes, and each construct contains three components downstream of the CAG promoter. For live-cell imaging of DNA double-strand-break (DSB) repair, we cloned a DNA fragment encoding the mouse 53BP1 foci-forming region (m53BP1FFR) fused to the fluorescent protein (FP) Ypet. For live-cell imaging of cell cycle-associated proteins, we cloned fragments of the human proteins hCdt1 (amino acids 30–120) and hGmnn (amino acids 1–110). In Focicle1 and Focicle2, hCdt1 was fused to mRuby3 and hGmnn was fused to mTagBFP2. The coding sequence of Focicle1 begins from Ypet/mBP1TD, whereas it begins from mRuby3 in Focicle2. In Focicle3, hCdt1 was fused to mTagBFP2 and hGmnn was fused to mRuby3. The three components of the constructs were connected by 2A peptides (P2A or T2A).

**(A)**


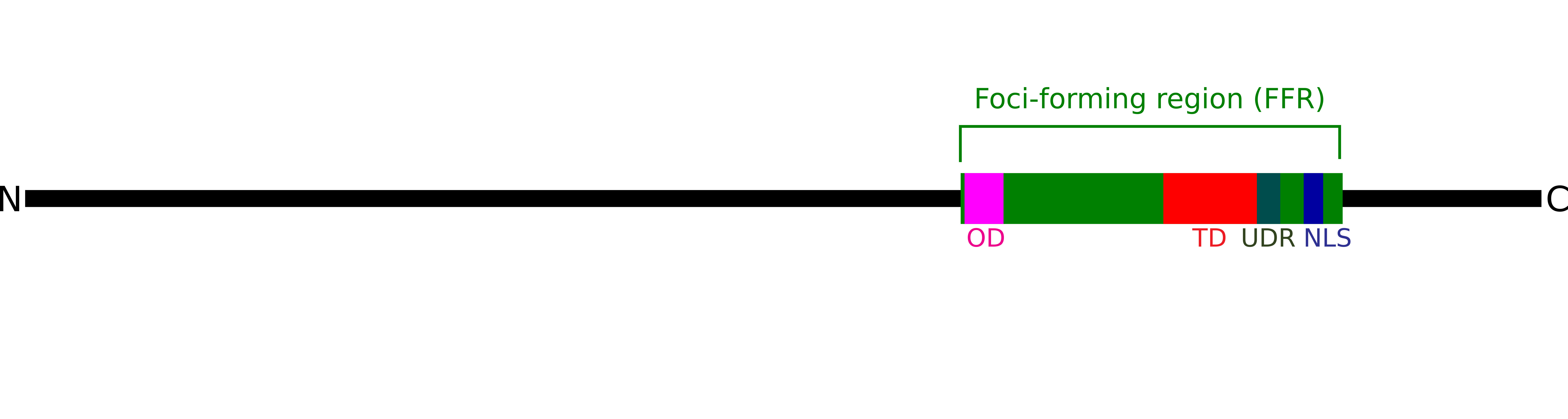


**(B)**

MPGEQMDPTGSQLDSDFSQQDTPCLIIEDSQPESQVLEEDAGSHFSVLSRHLPNLQMHKENPVLDIVSNPEQSAVEQGDSNSSFNEHLKEKKASDPVESSHLGTSGSISQVIERLPQPNRTSSALAVTVEAASLPEEEKEEEELEEEKEGVGANAPGADSLAAEDSASSQLGFGVLELSQSQDVEEHTVPYDVNQEHLQLVTTNSGSSPLSDVDASTAIKCEEQPTEDIAMIEQPSKDIPVTVQPGKGIHVVEEQNLPLVRSEDRPSSPQVSVAAVETKEQVPARELLEEGPQVQPSSEPEVSSTQEDLFDQSSKTASDGCSTPSREEGGCSPVSTPATTLQLLQLSGQKPLVQESLSTNSSDLVAPSPDAFRSTPFIVPSSPTEQGGRKDEPMDMSVIPVGGEPFQKLHDDEAMETEKPLLPSQPAVSPQASTPVSRSTPVFTPGSLPIPSQPEFSHDIFIPSPSLEEPSDDVKKGGGLHSSSLTVECSKTSESEPKNFTDDLGLSMTGDSCKLMLSTSEYSQSSKMESLGSPRTEEDRENTQIDDTEPLSPVSNSKLPADSENVLVTPSQDDQVEMSQNVDKAKEDETEDRGDCKGREDAVAEDVCIDLTCDSGSQAVPSPATRSEALSSVLDQEEAMDTKEHHPEEGFSGSEVEEVPETPCGSHREEPKEEPMESIPLHLSLTETQSEALCLQKEAPKEECPEAMEVETSVISIDSPQKLQVLDQELEHKDPDTWEEATSEDSSVVIVDVKEPSPRADVSCEPLEEVEKCSDSQSWEGVAPEEEPCAENRLDTPEEKRIECDGDSKAETTEKDAVTEDSPQPPLPSVRDEPVRPDQETQQPQVQEKESPVTVDAEVADDKQLGPEGACQQLEKAPACASQSFCESSSETPFHFTLPKEGDIIPPLTGATPPLIGHLKLEPKRHSTPIGISNYPESTIATSDVTSESMVEINDPLLGNEKGDSESAPEMDGKLSLKMKLVSPETEASEESLQFSLEKPTTAERKNGSTAIAEPVASLQKPVPVFGCIYEAQQEKEAQSEAPPSAPDRANLLHFPSAQEEDKERPDVTPKLRQSEQPVKPVGPVMDDAAPEDSASPVSQQRASQEQRASQEPFSPAEDVMETDLLEGLAANQDRPSKMLMDRPTQSNIGIQTVDHSLCAPETVSAATQTVKSVCEQGTSTAEQNSGKQDATVQTERGSGEKPASAPVDDTESLH**SQGEEEFEMPQPPHGHVLHRHMRTIREVRTLVTRVITDVYYVDGTEVERKVTEETEEPIVECQECETEVSPSQTGGSSGDLGDISSFSSKASSSHHTSSGTSLSAIHSSGSSGRGAGPLKGKASGTEAADFALPSSRGGPGKLSPRKGISQTGAPVCEEDGDAGLGIRQGGKAPVTPRGRGRRGRPPSRTTGTRETVVSGPLGVEDISPSMSPDDKSFTRIMPRVPDSTKRTDASSSTLRRSDSPEIPFQAATGSSDGLDSSSSGNSFVGLRVVAKWSSNGYFYSGKITRDVGAGKYKLLFDDGYECDVLGKDILLCDPIPLDTEVTALSEDEYFSAGVVKGHRKESGELYYSIEKEGQRKWYKRMAVILSLEQGNRLREQYGLGPYEAVTPLTKAADISLDNLVEGKRKRRSNISSPVTPTAASSSSTTPTRKATESPRASTGVPSGKRKLPTSEEERSPAKRGRKSATVKPGTVGAAEFVSPCETGDNIGE**PSVLEEPRGPLPLNKTLFLGYAFLLTMATTSDKLASRSKLLDGPTGSSEEEEEFLEIPPFNKQYTECQLRAGAGYILEDFNEAQCNTAYQCLLIADQHCRTRKYFLCLASGIPCVSHVWVHDSCHANQLQNYRNYLLPAGYSLEEQRILDWQPRENPFQNLKVLLVSDQQQNFLELWSEILMTGGAASVKQHHSSAHNKDIALGVFDVVVTDPSCPASVLKCAEALQLPVVSQEWVIQCLIVGERIGFKQHPKYKHDYVSH*

**Supplementary Fig S2**

Sequence of the mouse 53BP1 foci-forming region (m53BP1FFR). (A) Structure of mouse 53BP1 protein. OD, oligomerization domain; TD, Tudor domain; UDR, ubiquitylation-dependent recruitment motif; NLS, nuclear localization signal. (B) Amino acid sequence of mouse 53BP1. Highlights show sequences of the OD (purple, Zgheib et al. 2009), TD (red), UDR (green, Fradet-Turcotte et al. 2013), and the predicted NLS (blue). Our cloning sequence is shown in **bold** letters.

**(A)**


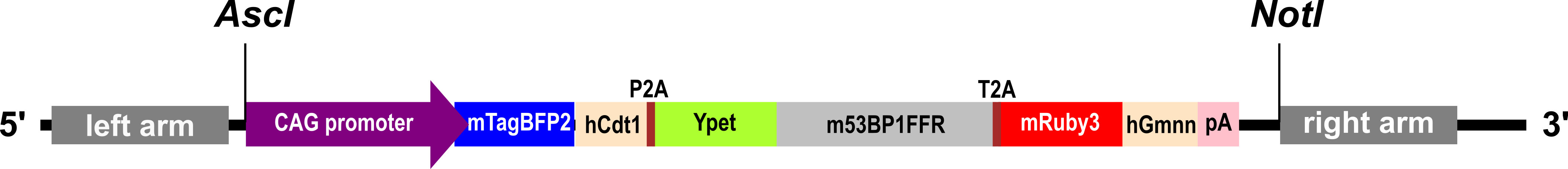


**(B)**


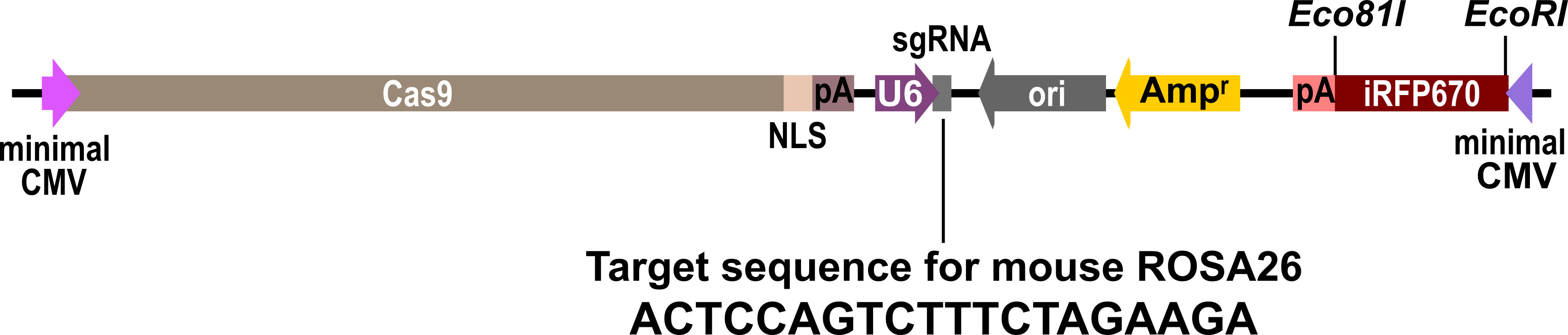


**Supplementary Fig. S3**

Design of plasmids for genome editing. (A) Focicle1 probe containing three fusion genes downstream of the CAG promoter was inserted into the pUC19 plasmid harboring mouse ROSA26 left and right arms. (B) The Cas9 expression vector harbored an sgRNA scaffold, the target sequence for the mouse ROSA26 locus, and the gene encoding the reporter iRFP670.


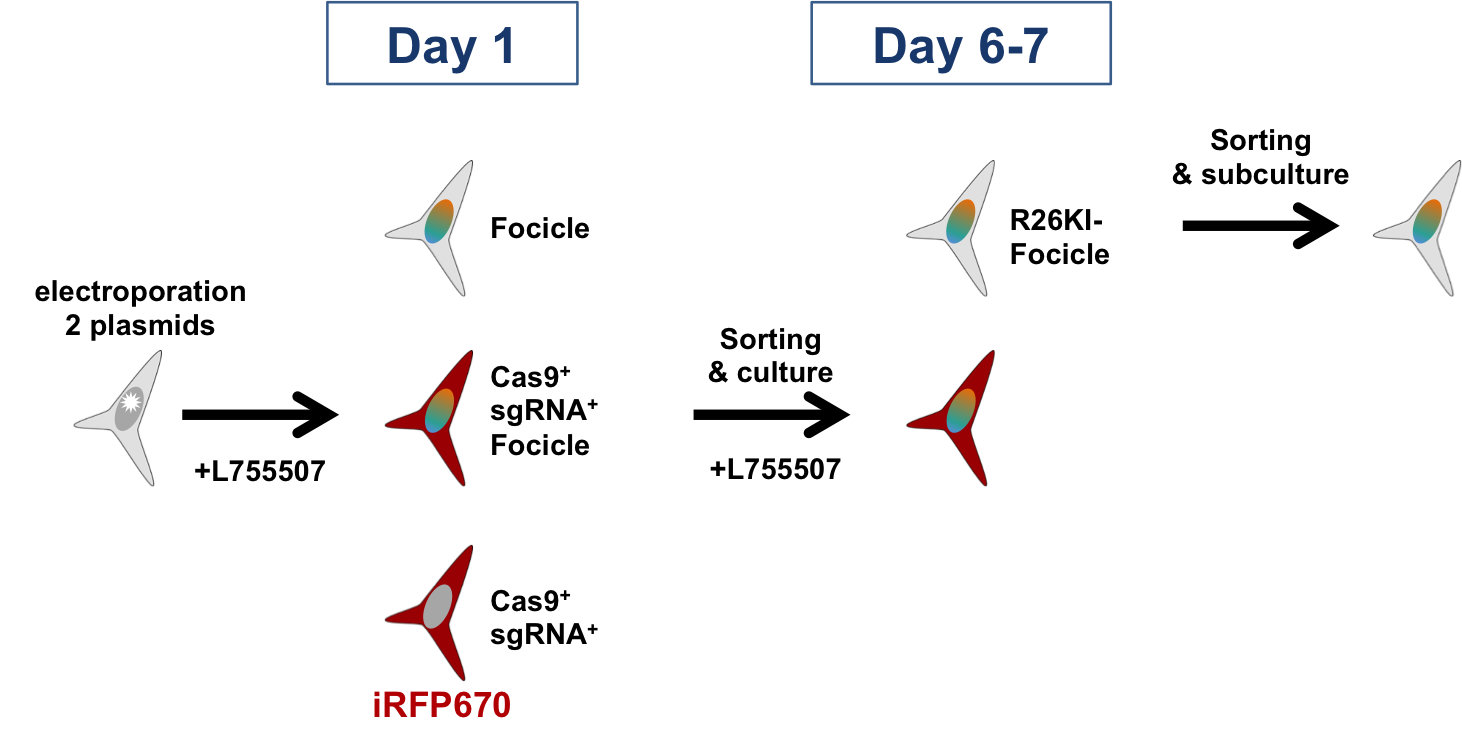


**Supplementary Fig. S4**

Scheme used for isolating knock-in cells. Two plasmids for genome editing (see Supplementary Fig. S3) were transfected into NIH3T3 cells through electroporation, and then the cells were incubated in medium containing L755507. On Day 1, Ypet^+^/iRFP670^+^ cells were isolated by means of cell sorting and incubated for approximately 1 week in medium containing L755507. To exclude Cas9-expressing cells, Ypet^+^/iRFP670^-^ cells were isolated through cell sorting and subcultured on Days 6–7.


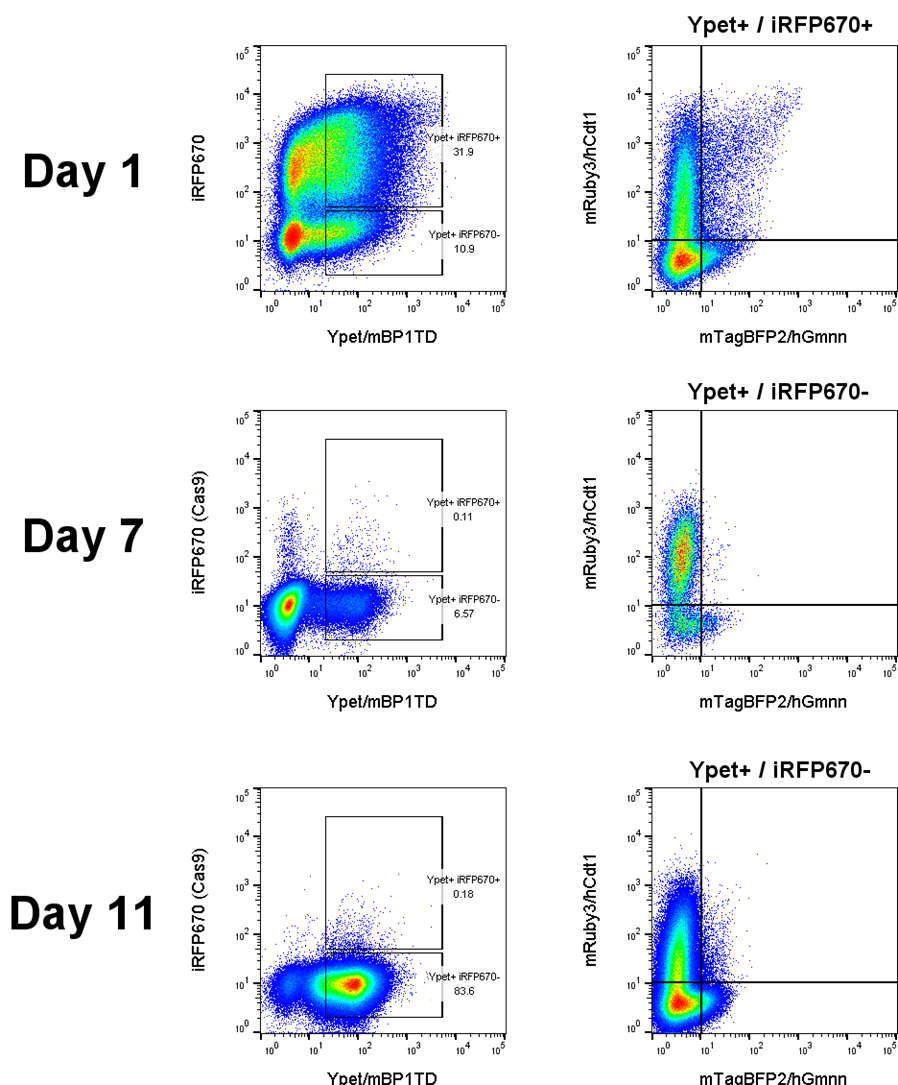


**Supplementary Fig. S5**

The fluorescent distribution of cells during the knock-in process. On day 1, it was possible to detect a population expressing both Ypet/mBP1TD and iRFP670 (Cas9). On day 7, the Ypet^+^/iRFP670^-^ population (about 5%) was isolated to exclude cells expressing Cas9 protein. Isolated cells continuing to express Ypet^+^ indicated that Focicle knock-in cells were obtained successfully (Day 11). Right panels show the fluorescent (mTagBFP2 and mRuby3) distribution of cells in the gate of Ypet^+^/iRFP670^+^, Ypet^+^/iRFP670^-^, Ypet^+^/iRFP670^-^ at Day 1, 7, and 11, respectively.

**
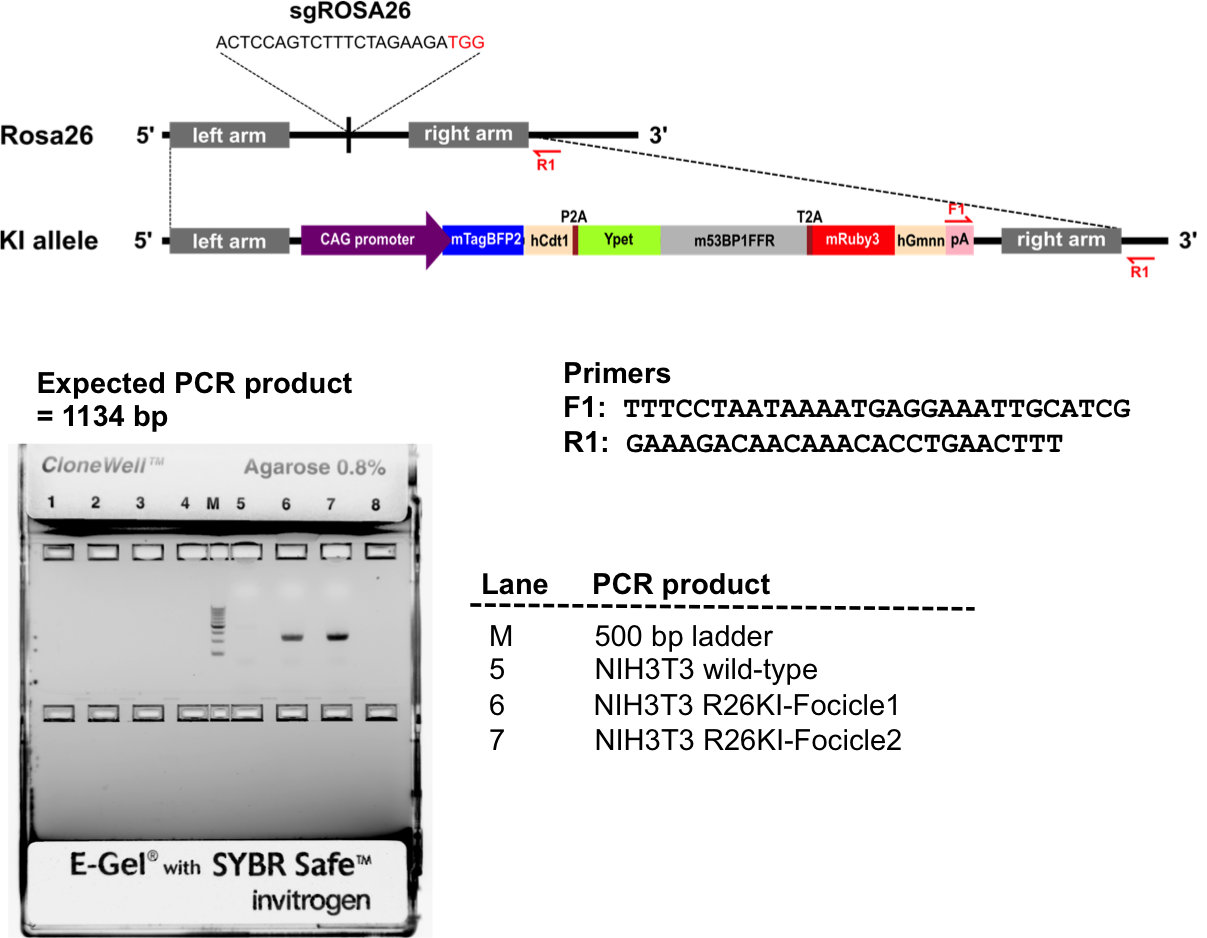
**

**Supplementary Fig. S6**

Validation of knock-in by PCR. Ypet^+^ cells of NIH3T3 cells harboring R26KI-Focicle1 and R26KI-Focicle2 were purified and their genomic DNA was extracted. PCR was performed with the forward primer (F1) and reverse primer (R1) as shown in Supplementary Fig. S5. Expected PCR products were observed only in the cells harboring R26KI-Focicle1 and R26KI-Focicle2.
